# Direct-Writing Electrospun Functionalized Scaffolds for Periodontal Regeneration: In Vitro Studies

**DOI:** 10.3390/jfb14050263

**Published:** 2023-05-09

**Authors:** Laura Bourdon, Nina Attik, Liza Belkessam, Charlène Chevalier, Colin Bousige, Arnaud Brioude, Vincent Salles

**Affiliations:** 1 Laboratoire des Multimatériaux et Interfaces, UMR 5615, CNRS, Université Claude Bernard Lyon 1, Bâtiment Chevreul, 6 Rue Victor Grignard, 69622 Villeurbanne, France; laura.bourdon@itech.fr (L.B.); nina.attik@univ-lyon1.fr (N.A.); belkessamliza@yahoo.com (L.B.); charlene.chevalier@univ-lyon1.fr (C.C.); colin.bousige@univ-lyon1.fr (C.B.); arnaud.brioude@univ-lyon1.fr (A.B.); 2 Faculté d’Odontologie, Université Lyon 1, 11 Rue Guillaume Paradin, 69008 Lyon, France; 3 LIMMS, CNRS-IIS UMI 2820, The University of Tokyo, Tokyo 153-8505, Japan; 4 Institute of Industrial Science, The University of Tokyo, Tokyo 153-8505, Japan

**Keywords:** direct-writing, electrospinning, bifunctional, scaffold, periodontal ligament cells, hydroxyapatite nanoparticles, CEMP1, cell mineralization, periodontal regeneration

## Abstract

Multiphasic scaffolds that combine different architectural, physical, and biological properties are the best option for the regeneration of complex tissues such as the periodontium. Current developed scaffolds generally lack architectural accuracy and rely on multistep manufacturing, which is difficult to implement for clinical applications. In this context, direct-writing electrospinning (DWE) represents a promising and rapid technique for developing thin 3D scaffolds with controlled architecture. The current study aimed to elaborate a biphasic scaffold using DWE based on two polycaprolactone solutions with interesting properties for bone and cement regeneration. One of the two scaffold parts contained hydroxyapatite nanoparticles (HAP) and the other contained the cementum protein 1 (CEMP1). After morphological characterizations, the elaborated scaffolds were assessed regarding periodontal ligament (PDL) cells in terms of cell proliferation, colonization, and mineralization ability. The results demonstrated that both HAP- and CEMP1-functionalized scaffolds were colonized by PDL cells and enhanced mineralization ability compared to unfunctionalized scaffolds, as revealed by alizarin red staining and OPN protein fluorescent expression. Taken together, the current data highlighted the potential of functional and organized scaffolds to stimulate bone and cementum regeneration. Moreover, DWE could be used to develop smart scaffolds with the ability to spatially control cellular orientation with suitable cellular activity at the micrometer scale, thereby enhancing periodontal and other complex tissue regeneration.

## 1. Introduction

Periodontitis is a prevalent inflammatory disease that damages the periodontal tissues [1] and is difficult to treat. Three tissues are degraded: the mineralized cementum that covers the tooth, the alveolar bone that supports it, and the periodontal ligaments that bind the two first tissues [2]. The current regenerative methods involve either a “guided tissue regeneration” membrane [3] or an “enamel matrix derivative” gel, also known as Emdogain^®^ [4,5]. In the first case, free spaces under the gum are created to stimulate regeneration [3], whereas, in the second case, amelogenin proteins are used to stimulate cellular mineralization [6]. Even though both methods have demonstrated positive outcomes, the results are often unpredictable [6,7,8]. Periodontal healing requires a well-coordinated regeneration of intricate mineralized and soft tissues that is difficult to achieve without scaffolding [9].

A recent trend consists of using multiphasic scaffolds for guiding the reconstruction of tissue complexes. They combine multiple compartments that are differentiated by their architectural, physical, or biochemical properties, with each one being adapted for regenerating a specific tissue [10,11]. For periodontal regeneration, the team of S. Sowmya et al. fabricated a scaffold with three layers of hydrogel containing distinctive bioactive properties that induced cementogenic, fibrogenic, or osteogenic differentiation of dental follicle stem cells [12]. Hydrogels are capable of encapsulating bioactive agents that stimulate cell differentiation while providing physical support for cell proliferation. However, the non-oriented porosity composing hydrogels is not adapted for regenerating oriented tissues such as periodontal ligament fibers. On the other hand, 3D printing and electrospinning techniques can fabricate fibrous scaffolds for tissue regeneration. In contrast to the former, which fabricates large constructs for regenerating the bone defect, the latter fabricates thin constructs with micrometric filaments that show promising results for guiding PDL regeneration [13,14,15,16].

Direct-writing electrospinning (DWE) has emerged as a promising fabrication technique. It combines the advantages of 3D printing for precise architectural control and electrospinning technologies for fabricating micrometric fibrous structures that mimic the natural extracellular matrix [17,18]. In this process, a micrometric filament is spun from a melt or polymer solution and subjected to an electric field. This filament is continuously deposited on a moving collector in a controlled pattern to form 3D constructs. Among others, P. D. Dalton et al. have contributed significantly to developing molten polymer technology by fabricating structures with controlled architectures and mechanical properties [19,20,21,22,23]. However, the molten process hinders the encapsulation of bioactive agents that promote cellular activities for regeneration. DWE using a polymeric solution, although poorly harvested, represents an alternative method for fabricating functional scaffolds loaded with bioactive agents [24,25].

In this study, we used the solution-based DWE technique for fabricating functional scaffolds that could regenerate the interface between the two periodontal mineralized tissues (cementum and alveolar bone) based on two compartments integrating two bioactive agents.

First, we used poly(lactic-co-glycolic acid) (PLGA) and polycaprolactone (PCL) polymers that have mechanical properties, biocompatibility, and a degradation rate suitable for tissue regeneration [10,26]. We integrated two bioactive agents that are commonly used for bone and periodontal regeneration: hydroxyapatite nanoparticles (HAP NPs) and Cementum 1 protein (CEMP1), respectively. HAP is a ceramic with a chemical composition close to bone, thus making it interesting for regeneration. It is also biocompatible, induces both osteoconduction and osteoinduction [27,28], and can promote PDL cell differentiation into osteoblast-like cells [29,30,31]. CEMP1 is expressed by periodontal cells and is involved in the regeneration process by differentiating PDL cells into a cementoblastic phenotype [32,33]. Since HAP and CEMP1 were reported to induce osteoblast-like and cementoblast-like cells from PDL cells, respectively, the two bioactive agents were integrated in the fibrous scaffold to simulate the in vivo regeneration of the two mineralized tissues. It has been recently reported that the HAP type, such as calcium-deficient hydroxyapatite, and the HAP preparation method, such as the hydrothermal process, could influence the mechanical and bioactivity of the final elaborated material [34,35]. In this respect, standardized commercial HAP nanoparticles that are commonly investigated to functionalize scaffolds for different medical applications were used in this study.

**Figure 1:**
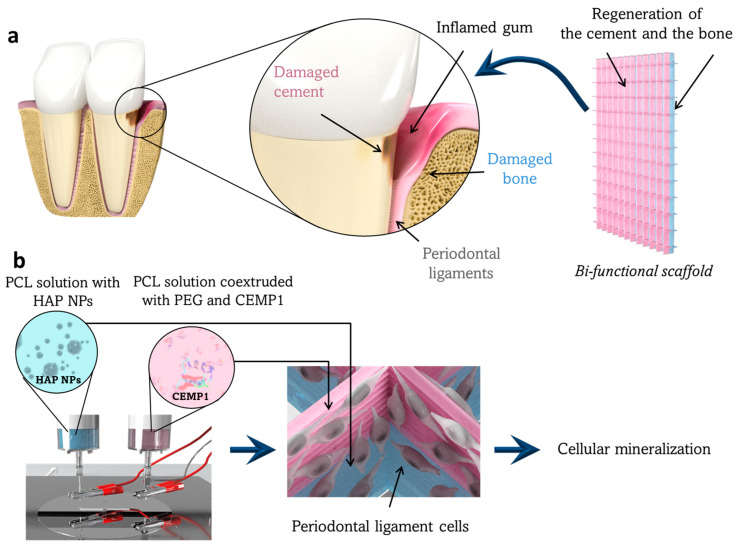
Working principle and elaboration process of the designed bifunctional scaffold for periodontal tissue regeneration. (**a**) Representation of a periodontal pocket lesion. This lesion can be filled by the bifunctional scaffold to regenerate the damaged cementum and alveolar bone tissues. This scaffold has two layers to regenerate the cementum from one side (in pink) and the bone from the other side (in blue). (**b**) The scaffold is fabricated by DWE in two steps with two spinnerets delivering one solution of HAP NPs (in blue) and one solution of CEMP1 (in pink). The periodontal ligament cells, which remain in the periodontal defect or are used to colonize the scaffold (image in the center). In contact with the scaffold containing the HAP (in blue) and the CEMP1 (in pink), the cells will thereafter differentiate into osteoblast-like and cementoblast-like cells, respectively.

As shown in Figure 1, we developed a bifunctional scaffold that combined HAP NPs for bone regeneration on one side of the scaffold and CEMP1 for cementum regeneration on the other side. The whole protocol is further illustrated in a video (Video S1). Then, the effects of the two obtained functional scaffolds (HAP-based and CEMP1-based) were evaluated independently, in vitro, on PDL cells’ behavior. To this end, cell proliferation and colonization were evaluated using Alamar Blue assay and confocal imaging, respectively. Moreover, mineralization potential was assessed through the quantification of mineralized nodules and osteopontin (OPN) expression, a protein naturally present in human mineralized tissues and commonly investigated as a mineralization marker [36,37].

## 2. Materials and Methods

### 2.1. Material

Polycaprolactone (PCL) and poly(lactic-co-glycolic acid) (PLGA) (with 85% of lactic monomer) with an inherent viscosity of 1.6 dL·g^−1^ and 2.3 dL·g^−1^, respectively, were purchased from CORBION (medical grade), while polyethylene glycol (PEG, M_w_ = 35 kDa) was purchased from Sigma Aldrich. The organic solvent 1,1,1,3,3,3-hexafluoro-2-propanol (HFP) was purchased from TCI. Dry HAP NPs with a diameter smaller than 200 nm and lyophilized BSA were purchased from Sigma Aldrich, and CEMP1 recombinant protein was purchased from CUSABIO.

### 2.2. DWE Set-Up

**Figure 2:**
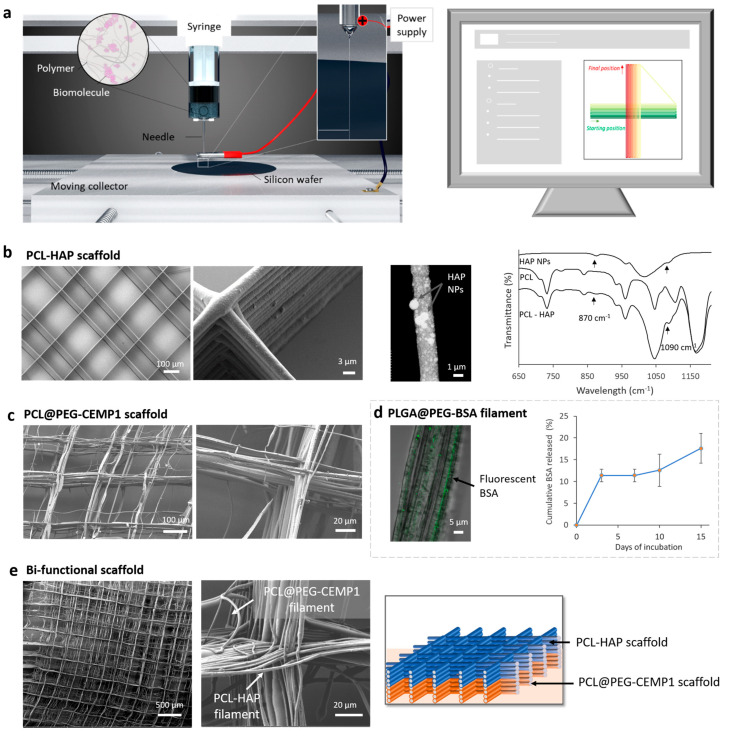
DWE fabrication and characterization of functional scaffolds. (**a**) Schematic representation of the DWE setup where solution flow rate, collector X-Y-Z, displacement speed, and voltage are controlled by computer. (**b**) On the left side, two SEM images of the PCL-HAP scaffold fabricated by DWE. In the center, STEM image of an isolated PCL filament with HAP NPs deposited by DWE. On the right side, FTIR ATR of HAP NPs, PCL, and PCL-HAP mats fabricated by electrospinning. (**c**) On the left side, two SEM images of the PCL@PEG-CEMP1 scaffold fabricated by DWE. (**d**) CLSM image of core-shell PLGA@PEG filaments containing fluorescent BSA in the core and deposited by DWE. On the right side, cumulative BSA released from core-shell PLGA@PEG filament mats in water. (**e**) Two SEM images of the bifunctional scaffold with PCL-HAP and core-shell PCL@PEG-CEMP1 parts. On the right side, schematic representation of the bifunctional scaffold.

Scaffolds were fabricated in a DWE setup (TOBECA) with temperature (20 ± 1 °C) and humidity (40 ± 1%) control. It contained a moving collector (in the X, Y, and Z axes) and two vertically oriented syringe pumps that delivered solutions through two needles independently connected to a power supply (Figure 2a).

### 2.3. Fabrication of the Scaffolds

An in-house software generated the coordinate codes that formed the scaffold pattern [38]. We chose to form a grid with 200 µm square pores that could provide good cell attachment and proliferation during the first 14 days of incubation [39]. The pattern was composed of 4 cm series of parallel lines alternatively printed with an angle of 0° and 90° (Figure 2a). The number of pattern repetitions tuned the number of stacked filaments (between 20 and 60 with the same solution). The scaffold was a 5 mm square resulting from the line cross-section.

The HAP-based scaffold was fabricated with a solution of 0.35 g·mL^−1^ PCL in HFP containing 30 wt% HAP NPs. The viscous solution was homogenized by stirring overnight and ultrasound bath for 30 min. For the fabrication, the solution was extruded through a 21 G needle with a flow rate of 0.02 mL·h^−1^.

The CEMP1-based scaffold was fabricated with coaxial needles supplied by one protein solution in the core needle and one polymeric solution in the shell needle. The solution of protein contained 2 mg·mL^−1^ CEMP1, 0.3 g·mL^−1^ PEG, 20 mM Tris-HCl, 0.5 M NaCl, and 10% glycerol, while the polymeric solution contained 0.35 g·mL^−1^ PCL in HFP. Considering the diameter of the core (28 G) and shell needles (21 G), the two solutions were extruded with a flow rate ratio of 4:1 (shell:core) to have similar extrusion speeds through both needles, i.e., 0.02 mL·h^−1^ and 0.005 mL·h^−1^, respectively.

Both scaffolds were fabricated on a silicon wafer with a voltage between 2 and 2.1 kV, a needle tip-to-collector distance of 3 mm, and collector speed of 25 cm·s^−1^.

### 2.4. Characterization of the Raw Scaffolds

Electronic microscopy: The morphology of scaffolds fabricated on silicon wafer was characterized by scanning electron microscopy (SEM) (Merlin Compact from Zeiss, Jena, Germany) after being coated with copper by sputtering (Baltec MED 020).

The distribution of HAP NPs in PCL filaments was observed by scanning transmission electron microscopy (STEM, HD-2300 from Hitashi). For that purpose, a filament was deposited by DWE on a TEM grid.

Chemical characterization: Fourier transform infrared spectroscopy with attenuated total reflectance (FTIR-ATR) (SAFAS) was used to characterize PCL filaments containing HAP NPs. Spectra from 4000 to 600 cm^−1^ were recorded with a resolution of 4 cm^−1^ and an average of 10 scans.

### 2.5. Scaffold Preparation before Cell Contact

After fabrication, the scaffolds were easily detached from the silicon wafer (see Figure S4d) by adding several drops of ethanol on the HAP-based scaffold and deionized water on the CEMP1 one; ethanol was avoided to preserve the protein. The scaffolds were dried for 6 days at 40 °C and 37 °C, respectively, in order to remove the remaining solvent. Thermal analysis revealed that less than 0.6% of solvent remained in the scaffolds after drying” under (2.5 scaffold preparation before cell contact). Thermal analysis revealed a weight loss of about 0.6% (Figure S1). This gaseous evolution could be due to loss of water or may be HFiP traces. We considered this ratio to be acceptable to proceed to the biological assays. The scaffolds were placed on a Thermanox^TM^ coverslip, introduced in wells of a 24-microwell plate, and maintained in the well bottom with sterilized PTFE gaskets. The plates were sterilized under 214 nm UV light for 40 min. For biological assays, the sample was triplicated.

### 2.6. Cell Culture

Human periodontal ligaments (PDLs) derived from human primary cell culture of the periodontal ligament tissue (#2630-sc, CliniSciences, Nanterre, France) were cultured in Fibroblast Medium, with 10% fetal bovine serum, 2% penicillin/streptomycin, and 1% amphotericin B. Cultures were maintained at 37 °C under a humidified atmosphere (5% CO_2_). After reaching confluence, the cells were trypsinized and maintained at 37 °C for further experiments. For every assay, PDL cells at 2 or 3 passages were used.

### 2.7. Assessment of Cell Biological Behavior

Cell cytotoxicity: Cell viability was characterized using a LIVE/DEAD™ Viability/Cytotoxicity Kit, (L3224, Invitrogen™ by Thermo Fisher Scientific, Waltham, MA, USA) after 7 days of culture in contact with the bifunctional scaffold. Living cells were identified by calcein AM green staining. Damaged cells were labeled (red) with ethidium homodimer (EthD-1). A working solution prepared by combining the two staining reagents was added to the tested samples at 37 °C for 5 min. The samples were washed with D-PBS and observed with confocal laser scanning microscopy (CLSM) (LEICA SP5 X Leica, Wetzlar, Germany).

Cell proliferation: Cell proliferation was determined through the metabolic activity quantified using an Alamar Blue assay (Alamar Blue^®^ solution, DAL1025, Thermo Scientific France, Illkirch-Graffenstaden, France). The assay was carried out using the modification method of McNicholl et al. [40]. For this, a 24-microwell plate was used as a ‘feeder tray’ in which 1 mL of 10^4^ cells·mL^−1^ cell suspension was seeded over the surface of the above scaffolds (direct contact) for 1, 3, 5, and 7 days. Unexposed control cells were maintained in the same conditions. The Alamar Blue solution was poured directly into the wells at the final concentration of 10% (*v/v*), and the plates were then incubated at 37 °C for 5 h. The amount of Resorufin formed was determined by measuring the absorbance at 570 and 600 nm using a micro-plate reader (Infinite^®^ M 200 PRO, NanoQuant plate, Tecan, France). Each sample was tested in triplicate, and two measurements were performed for each. The results were expressed as a percentage of cell viability of the untreated control (100%).

Cell colonization: Cell colonization was evaluated by CLSM. The samples were fixed with 3.7% formaldehyde solution in PBS for 30 min before washing with PBS. The cells were permeabilized with 1% Triton X-100 in PBS (5 min) and blocked with 1% bovine serum albumin (BSA) in PBS. Actin microfilaments were stained with Alexa Fluor 488 (AF488) phalloidin (green fluorescence), and cell nuclei were stained with propidium iodide (red fluorescence).

Detection and quantification of mineralized nodules: Alizarin Red S staining was used to detect mineralized nodules synthesized by the cells. The latter were initially seeded on the samples with a density of 10^3^ cells·mL^−1^. After 18 days of contact, cellular scaffolds were fixed using formaldehyde (3.7% in PBS, 30 min) and washed with deionized water. A total of 40 mM of Alizarin Red (pH 4.2) was added. The cells were incubated at room temperature for 40 min, then washed with deionized water three times and observed under an optical microscope (CKX41/CKX3, Olympus Microsystems, Rungis, France). The calcium amount quantification obtained from the synthesized mineralized nodules after Alizarin staining by dissolving them in a solution of 10% cetylpyridinium chloride (Sigma-Aldrich, St. Louis, MI, USA) for 15 min. The optical density representing the relative quantity of mineralization nodules was measured at 560 nm. Statistical analysis:

OPN expression: OPN expression was qualitatively assessed by immunostaining after 18 days of cell culture. OPN was stained with a rabbit antiosteopontin polyclonal antibody and ALEXA FLUOR^®^ 555 Conjugated (AF555, bs-0019R-A555, CliniSciences, France), and cell nuclei were stained using DAPI, (Cat# D1306, Thermo Scientific France) and the samples were observed by CLSM.

The data were reported with the standard deviation (±SD), and the statistical analyses were performed using the Student’s *t*-test.

## 3. Results

### 3.1. Preparation of the Bifunctional Scaffold

The encapsulation of proteins in scaffolds is challenging, as they can be easily denaturated by external stresses such as temperature modification, organic solvents, or mechanical stress [41,42,43]. In our study, we showed that the HFP solvent, used to dissolve resorbable polymers, modified the conformation of a model protein (BSA) by presuming the degradation of the protein of interest (CEMP1) (see Supplementary Material S1 and Figure S2). To preserve the protein, we isolated it from the resorbable polymer and the organic solvent by using a coextrusion system. The technique, called coaxial electrospinning, aims at forming core-shell filaments by extruding one solution in the core of another polymeric solution, which acts as a shell. The protein was blended in a hydrophilic polymer, polyethylene glycol (PEG), which helps to maintain the conformation of proteins in the solution (see Supplementary Material S1 and Figure S2) as well as in solid-state [44,45,46,47]. The HAP was encapsulated in the filament by blending HAP NPs inside the polymeric solution.

Two resorbable polymers, PLGA and PCL, were investigated to fabricate the scaffolds. We used a 0.35 g·mL^−1^ PCL solution and a 0.13 g·mL^−1^ PLGA solution in the HFP that both showed similar characteristics in conventional electrospinning. The jet pathway between the needle tip and the collector was stable and rectilinear until 15 to 20 cm of distance, thus presuming a high precision of direct-writing of the filament. Interestingly, the two solutions had different behaviors in DWE. While the PCL jet was directed onto the moving collector for more than 1 h without visible evolution, the PLGA jet rapidly evolved. After 5 to 10 min, the PLGA cone distorted (Figure S3) leading to jet deviation and a loss of deposition precision. Based on this technical limitation, only the PCL solution was used for the scaffold fabrication. We adjusted the collector speed displacement to draw straight lines with the deposited filament. When the PCL solution was spun on the collector moving at speeds lower than 10 cm·s^−1^, the filament formed coiled patterns (Figure S4a). At 10 cm·s^−1^, the filament followed the linear displacement of the collector perfectly, and, from 10 to 20 cm·s^−1^, the jet was more stretched, which resulted in the thinning of the deposited filament. In fact, in general, we observed that a higher stretching also meant that the jet dried more quickly. With a collector speed of 20 cm·s^−1^, homogeneous scaffolds were printed (Figure S4b,c).

The HAP-based scaffold was fabricated by DWE with the PCL solution supplemented with 30 wt% of dispersed HAP NPs. The functional filaments with 4 to 5 µm diameters (Figure S5) were deposited and stacked upon each other to form the 3D grid (Figure 2b). The filaments contained a high density of well-distributed HAP particles. Most of the particles had a diameter of less than 100 nm, but particles and/or agglomerates with a diameter up to 1 µm were also observed emerging from the filament’s surface. The presence of HAP at the surface was confirmed by FTIR analysis showing the characteristic peaks of HAP [48,49].

The CEMP1-based scaffold was fabricated using a coaxial needle that was supplied with the PCL solution, as well as the PEG solution containing the CEMP1. The resulting scaffold was composed of filaments with 2 to 3 µm in diameters (Figure S5), which were partially stacked upon each other. Although gaps of about ±50 µm were observed between the walls, the scaffold still formed a 3D grid (Figure 2c). In parallel to the scaffold fabrication, we proved that a model protein (BSA) could be encapsulated and released from core-shell filaments fabricated with the coaxial needle (Figure 2d). Interestingly, a higher amount of protein was released after 15 days of incubation from the core-shell filaments compared to conventional electrospun filaments (Figure S2).

The biphasic scaffold was finally fabricated in one step by superimposing the two previous HAP and CEMP1 scaffolds. A total of 30 filament layers were printed for each functional part, resulting in a biphasic scaffold of 60 partially stacked layers (Figure 2d).

### 3.2. Biological Behavior of Periodontal Cells in Contact with the Experimental Scaffold

The objective of this investigation was to assess the potential of the bifunctional scaffold to regenerate the two mineralized periodontal tissues. As a first step, the bifunctional scaffold effect on PDL cell cytotoxicity and cell metabolic activity was evaluated. It was cytocompatible with PDL cells, as it did not affect cell cytotoxicity and cell metabolic activity. A high number of living cells was also detected on the scaffold after 7 days of culture (Figure S6), which indicated no cytotoxic effect. As a second step, HAP-based and CEMP1-based scaffolds were evaluated separately to distinguish HAP particles and CEMP1 impact in terms of cell proliferation and remineralization (mineralized nodules and OPN expression).

#### 3.2.1. HAP-Based Scaffold

**Figure 3:**
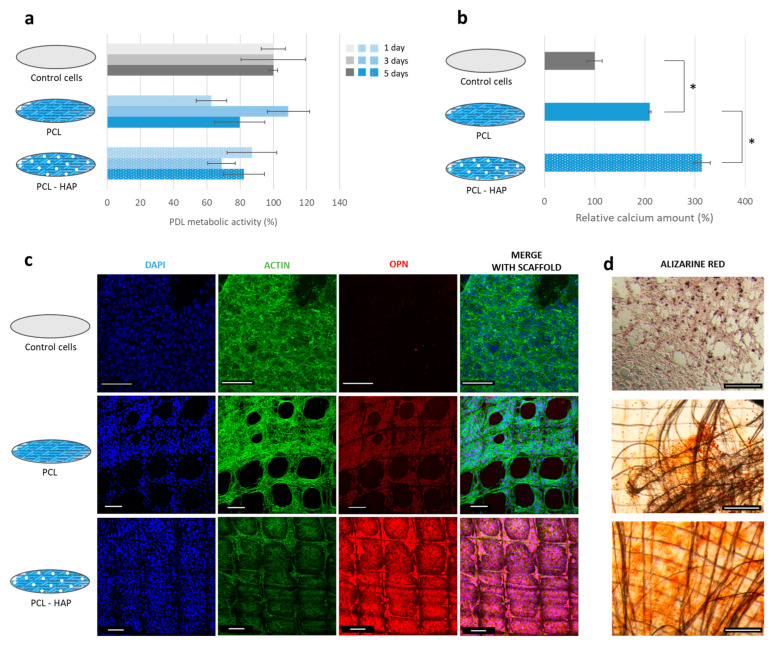
Biological evaluation of the HAP-based scaffold for bone regeneration. (**a**) PDL cell metabolic activity after 1, 3, and 5 days of direct contact. (**b**) Relative amount of calcium stained by Alizarin S Red in samples. * *p* < 0.001. (**c**) CLSM images of the samples: the cell nuclei were stained with DAPI (in blue), the OPN was labeled with an antiosteopontin conjugated with AF555 (in red), and the actin filaments were stained with phalloidin conjugated with AF488 (in green). (**d**) Optical images of the samples after staining the calcium with Alizarin S Red. All scale bars correspond to 100 µm.

After elaboration, the HAP-based scaffold was placed in contact with PDL cells. During the first 5 days of culture, PDL proliferation was not affected by the presence of the scaffold or the HAP, which suggests that the functional scaffold was cytocompatible (Figure 3a).

After 18 days of culture, a higher number of mineralized nodules was observed in wells containing this scaffold (Figure 3d). On the functional scaffold, nodules were uniformly distributed on the filament’s surface as well as between the walls of the scaffold. In contrast, a lower number of nodules was present on the unfunctionalized scaffold. The nodules mainly appeared on the edge of the scaffold where a high density of filaments crossed. The difference in the number of nodules between the two samples was confirmed by calcium content: 50% more calcium was detected in the PCL-HAP scaffold samples compared to unfunctionalized PCL (Figure 3b). The cells also expressed more OPN in contact with PCL-HAP than PCL (Figure 3c). This result indicates that the functional scaffold could enhance the PDL cell remineralization.

#### 3.2.2. CEMP1-Based Scaffold

CEMP1-based scaffolds were introduced in culture wells after preparation. Based on the scaffold dimensions, the amount of encapsulated protein in each sample was estimated at around 0.3 ng. To confirm the effect of the released CEMP1 on cells and discriminate it from the influence of the scaffold, the behavior of the cells was analyzed by both direct and indirect contact.

Direct contact: Four samples were prepared: (i) without scaffold or CEMP1 (Control cells), (ii) without scaffold but with CEMP1 in the culture medium (CEMP1 in solution), (iii) with scaffold without CEMP1 (PCL@PEG), and (iv) with scaffold containing CEMP1 (PCL@PEG-CEMP1). Since 0.3 ng of CEMP1 was encapsulated in the scaffold, the same amount was introduced in the culture medium “CEMP1 in solution”.

**Figure 4:**
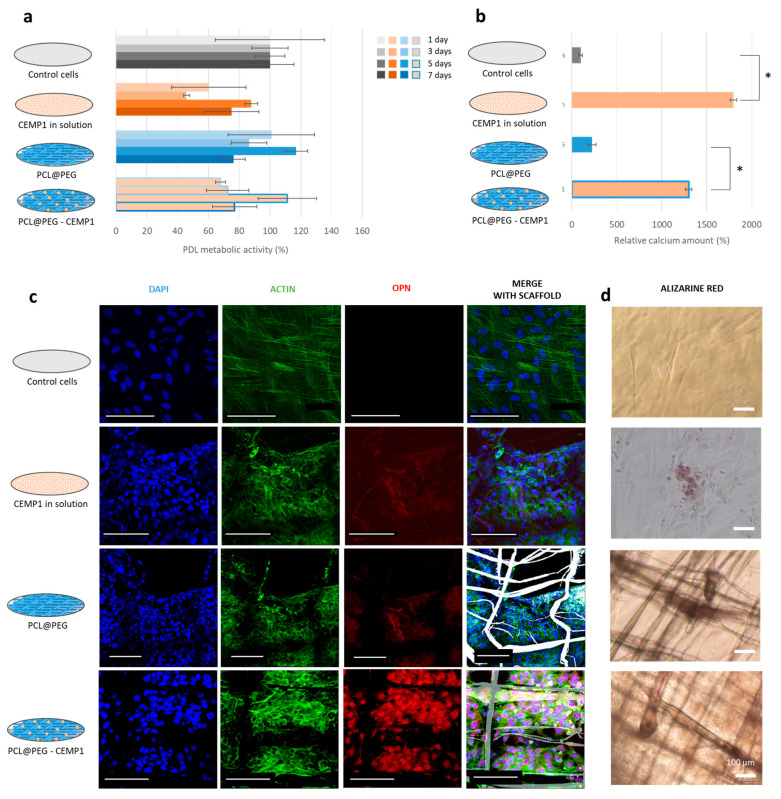
Biological evaluation of the CEMP1-based scaffold. (**a**) PDL cell metabolic activity after 1, 3, 5, and 7 days of culture. (**b**) Relative amount of calcium stained by Alizarin S Red in the samples. * *p* < 0.001. (**c**) CLSM images where the cell nuclei were stained with DAPI (in blue), the OPN was labeled with an antiosteopontin conjugated with AF555 (in red), the actin filaments were stained with phalloidin conjugated with AF488 (in green), and the scaffold appeared with bright field (in gray). (**d**) Optical images of the samples after staining by Alizarin S Red. All scale bars correspond to 100 µm.

During the first 7 days of culture, PDL cells had similar metabolic activity in the four sample types (Figure 4a). Cell proliferation was not affected by the presence of CEMP1 or by the scaffolds. However, the cells seemed to show favorable interactions with the two scaffolds by adhering to the surface of the filaments (Figure S7); cells had an elongated shape oriented in the direction of the filament.

After 18 days of culture, mineralized nodules were observed on the sample containing the CEMP1 in solution and the functional scaffold PCL@PEG-CEMP1 (Figure 4d); an amount of 15 times more calcium was significantly detected in them compared to samples without CEMP1 (Figure 4b). Confocal observation also revealed a higher level of OPN expression in the samples containing CEMP1 (Figure 4c). This shows that both CEMP1 in solution and the functionalized scaffold stimulated the cells to synthesize a mineralized matrix. The results suggest that the CEMP1 encapsulated in the scaffold could be released and that it influences the PDL cell behavior.

Indirect contact: To confirm that the CEMP1 was released from the functional scaffold, indirect contact tests were performed. For this, the CEMP1 in solution and the two types of scaffolds (PCL@PEG and PCL@PEG-CEMP1) were incubated for 14 days at 37 °C in the culture medium. PDL cells were then placed with each of these distinct eluates.

For the four conditions, there was no significant difference in metabolic activity of cells after 7 days in the eluates (Figure S8a). However, after 18 days, around three times more calcium was significantly detected (Figure S8b) and a higher OPN expression was observed on the PCL@PEG-CEMP1 sample compared to the control and to the PCL@PEG sample (Figure S8c). This could suggest that released CEMP1 from the scaffold remains bioactive, which thus resulted in an expected stimulated cell behavior.

## 4. Discussion

Through this study, we demonstrated the potential of DWE for developing multiphasic scaffolds with bioactive agents and a controlled microscopic architecture.

Our initial aim was to use two resorbable polymers in the scaffold—PCL and PLGA—that have biocompatible and resorbable properties suitable for guiding tissue regeneration [50,51]. Their combination in a multiphasic scaffold could be used to adjust the degradation rate and the kinetics of bioactive agent release according to the targeted tissue. The PCL solution was direct-written in a stable way, while the PLGA solution was easily destabilized, which makes it unusable in the fabrication of scaffolds. In fact, the destabilization was caused by an obvious drying of the solution at the needle tip. The right balance between avoiding early drying at the needle tip and the deposition of solid filaments could be found by adjusting the solvent type [52], the air humidity [53], or by using a system that limits the solution drying [54].

Moreover, this study aimed at showing the fabrication of functional filaments by DWE. Although scaffolds with HAP particles were previously made using DWE [55,56,57], these homogenous structures with stacked filaments contained a low quantity of HAP NPs. Other structures encapsulating a higher content of particles, as in our study, presented a heterogeneous architecture where the filaments did not stack accurately [56]. Here, we were able to develop a homogenous structure containing a high content of HAP NPs. So far, CEMP1 encapsulation via electrospinning has been rarely reported. The team of Chen et al. [58] fabricated an electrospun mat containing CEMP1 but the protein was encapsulated in mineral particles dispersed in the solution before electrospinning. In the current study, we directly encapsulated the protein during the filament fabrication, which simplifies the procedure and hinders protein loss. With a coextrusion system, which is rarely used in DWE, we encapsulated the protein in a protective environment, thereby isolating it from the resorbable polymer and organic solvent.

Furthermore, we fabricated one of the first biphasic scaffolds by integrating two bioactive agents using DWE. The current scaffold had two parts composed of 60 filament layers corresponding to an approximate height of 200 to 250 µm. The printed structures did not always fit with the theoretical pattern, especially for the CEMP1-based scaffold. Filaments were precisely stacked on each other due to an electrostatic attraction between the deposited filament and each previous layer [59]. The stacking capacity is limited when the electrostatic charges on the deposited filament are not evacuated through the grounded collector [59,60]. In our case, the CEMP1-based filament probably maintained a high amount of electrostatic charges after deposition, which implied partial stacking. The deposition resolution could be retrieved with further optimization, such as adjusting the two solution concentrations or by adjusting the voltage [60]. Additionally, these adjustments could increase the structure height to the millimeter scale, thereby expanding design optimization options. In the current study, the height of the structure was chosen to roughly correspond to the thickness of the alveolar ligament, which is typically in the range of 0.15–0.25 mm. The size of the X–Y shape was 5 cm square for practical reasons. However, as the scaffold was fabricated by computer-aided design, it could be easily adjusted over several centimeters according to the periodontal defect.

The study was followed by confirming the potential of the biphasic scaffold for periodontal regeneration. As a proof of concept, PDL cell proliferation, colonization, and remineralization ability on both independent HAP- and CEMP1-based scaffolds were assessed in vitro.

The experimental functionalized scaffolds stimulated PDL cell mineralization ability, as it was highlighted by an OPN protein expression increase, a larger number of mineralized nodule detections, and a higher amount of calcium deposition. The enhanced cell remineralization shows that both encapsulated HAP particles and CEMP1 protein influenced the cell behavior. Cells could interact with HAP by direct contact with the particle emerging from the filament. The encapsulation of particles in filaments produced a rough surface that could also influence the cell behavior [61]. It has been established that the material surface roughness influences cell adhesion and spreading, and could, thus, influence the biocompatibility and, consequently, osteointegration in vivo [62,63]. This is in agreement with the current findings, which demonstrated the enhancement of PDL cell colonization and mineralization potential in contact with the obtained HAP-based scaffold rough surfaces. The CEMP1 significantly enhanced the cell behavior even at low concentrations, which confirms that the protein was correctly encapsulated and released from the scaffold. The resulting porous structure in the current developed scaffold allows for physiological fluid and nutrient diffusion, in addition to the cell interconnection, despite the absence of a vascular system [64], which could contribute to the enhancement of the regenerative potential of the developed scaffold. Imber et al. suggested a pore size around 100 µm to be ideal for PDL regeneration [65]. In the current study, the pore size was about 200 µm.

Considering the enhanced cell colonization and mineralization behavior and that HAP and CEMP1 are known for inducing PDL differentiation [30,32], we assumed that the periodontal cells differentiated into cementoblast-like cells on the CEMP1-based scaffold and into osteoblast-like cells on the HAP-based scaffold. This hypothesis would need to be confirmed with additional cellular assessment, such as the expression level of OCN, CAP, and CEMP1 biomarkers [12,32,58]. In case the hypothesis is confirmed, fabricating scaffolds with DWE could represent a promising new approach for periodontal regeneration.

Despite the absence of topographical and mechanical scaffold characterization and in vitro assessment limitations in predicting the PDL tissue complexity in vivo, the obtained results provide relevant insight on the development of the functionalized scaffold. To the authors’ best knowledge, this is the first study investigating the use of the DWE method combined with conventional electrospinning for functionalized scaffold development for periodontal regeneration. The current obtained data could be correlated to both mechanical and future topographical surface properties to achieve a complete assessment with the aim of understanding how these parameters could influence the biological behavior of PDL cells.

Further optimizations could be suggested such as encapsulating additional proteins such as amylogenic peptides for cementum regeneration, CTGF, or fibroblast growth factor for PDL regeneration and BMP-2, or platelet-rich plasma derived growth factor, to further enhance the bioactivity [10,11,12,66]. The scaffold design could also be improved by integrating a third compartment for guiding the regeneration of PDL fibers binding the two mineralized tissues, the cementum, and the bone. The size and shape of the scaffold porosity could also be adjusted to optimize cell bioactivity and biocompatibility [67,68,69]. Finally, another advantage of the DWE setup is the possibility of performing conventional electrospinning by simply increasing the working distance. In that manner, intermediate layers with a smaller pore could be formed between scaffold compartments to decrease their permeability and control the diffusion of the contained bioactive compounds.

## 5. Conclusions

A bifunctional scaffold was developed using direct-writing electrospinning for regenerating the attachment interface between the two periodontal mineralized tissues: cementum and alveolar bone. The scaffold architecture was successfully controlled by producing a grid with 200 µm pores and 60 layers of stacked micrometric filaments. By adapting the DWE setup, HAP NPs and the CEMP1 protein were encapsulated. Finally, a bi-functional scaffold was developed with two compartments containing HAP NPs on one side for bone regeneration and CEMP1 on the other for the cementum regeneration.

Both independent functionalized scaffolds enhanced the PDL cells remineralization ability as compared to the unfunctionalized scaffold as demonstrated by extracellular calcium deposition and the OPN osteogenic marker overexpression. Moreover, the entire scaffold was colonized with cells; this underlines that the two bioactive agents encapsulated in the scaffold (e.g., HAP NPs and CEMP1) were able to stimulate and enhance the targeted cell activity.

Combining the DWE high resolution and the possibility of integrating multiple bioactive agents opens new perspectives for multi-tissue regeneration. The fibrous and functional architecture allows for suitable cellular orientation, tissue growth, and cellular activity in a spatially controlled manner at the micrometer scale. DWE arises from conventional electrospinning and 3D printing, two technologies which are easily accessible, and could provide controlled processes and predictable models with potential use for large-scale fabrication.

## Data Availability

All data are available upon request from the corresponding author.

## Supplementary Materials

The following supporting information can be downloaded at: https://www.mdpi.com/article/10.3390/jfb14050263/s1, Video S1: working principle; Supplementary Material S1: optimization of scaffolds containing protein; Supplementary figures.
