# Supplementary material for: Direct-Writing Electrospun Functionalized Scaffolds for Periodontal Regeneration: In Vitro Studies
Source: J Funct Biomater. 2023 May 9;14(5):263. doi: 10.3390/jfb14050263 (PMC10218971; doi:10.3390/jfb14050263)
Supplement: Supplementary file 1 [file jfb-14-00263-s001.zip › Supplementary figures.pdf]

SUPPLEMENTARY FIGURES

Direct-Writing Electrospun of Functional Scaffolds for Periodontal Regeneration: In Vitro Studies

Laura Bourdon[a], Nina Attik[a,b], Liza Belkessam[a,b], Charlène Chevalier[a,b], Colin Bousige[a], Arnaud Brioude[a] and Vincent Salles[a*]

[a] Laboratoire des Multimatériaux et Interfaces, UMR 5615, CNRS, Université Claude Bernard Lyon 1, Bâtiment Chevreul, 6 rue Victor Grignard, 69622 Villeurbanne, France.

[b] Faculté d'Odontologie, Université Lyon 1, 11 Rue Guillaume Paradin, 69008 Lyon, France

*Corresponding author: Vincent Salles, Associate-Professor of Lyon University (vincent.salles@univ-lyon1.fr) and visiting researcher in the University of Tokyo

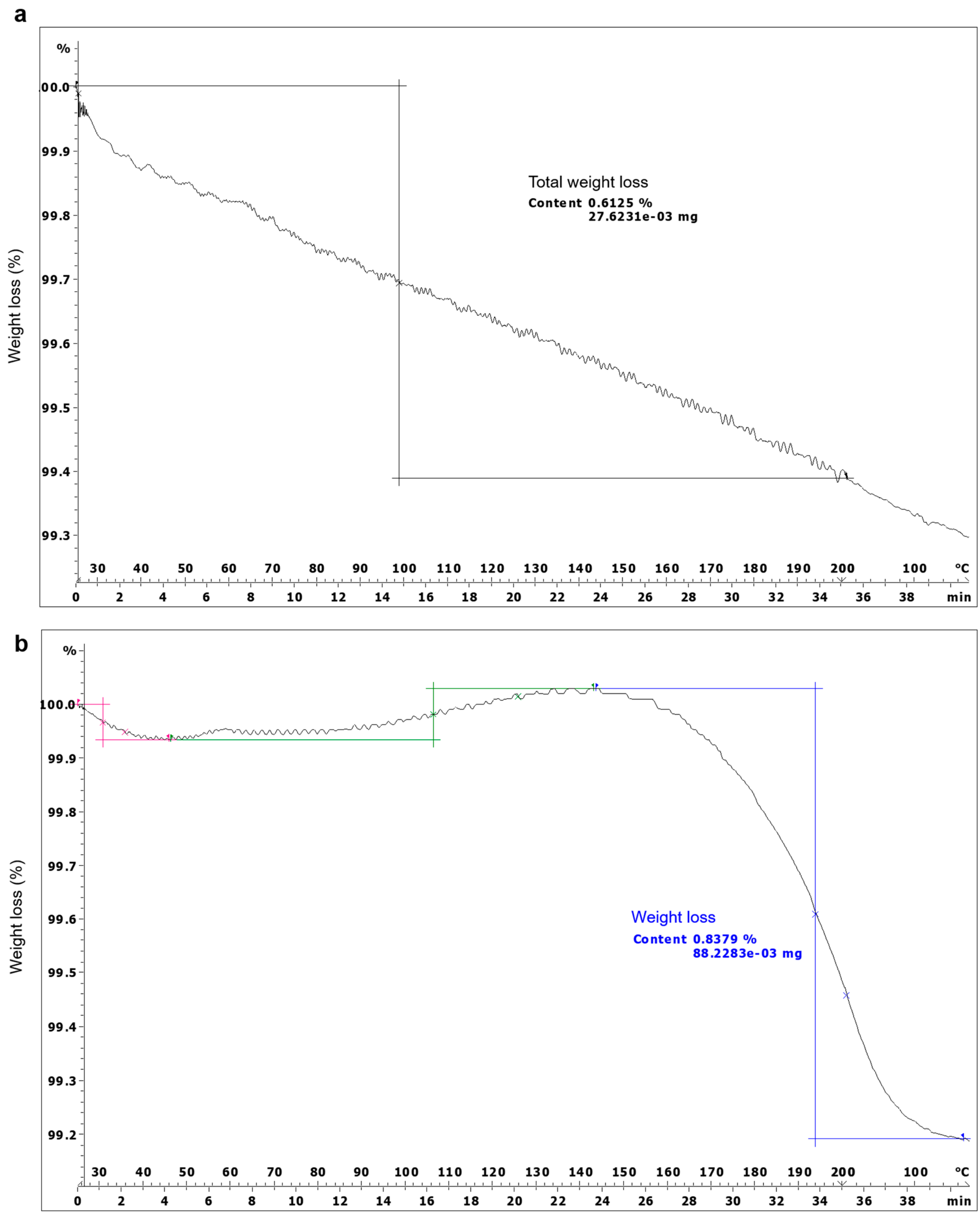

Figure S1. Thermogravimetric analysis (TGA/DSC3+, Mettler-Toledo) of a) PCL scaffold and b) PCL@PEG scaffold after the drying process. The weight loss was plotted during heating from 25 to 200°C under air at 5°C.min^{-1}.

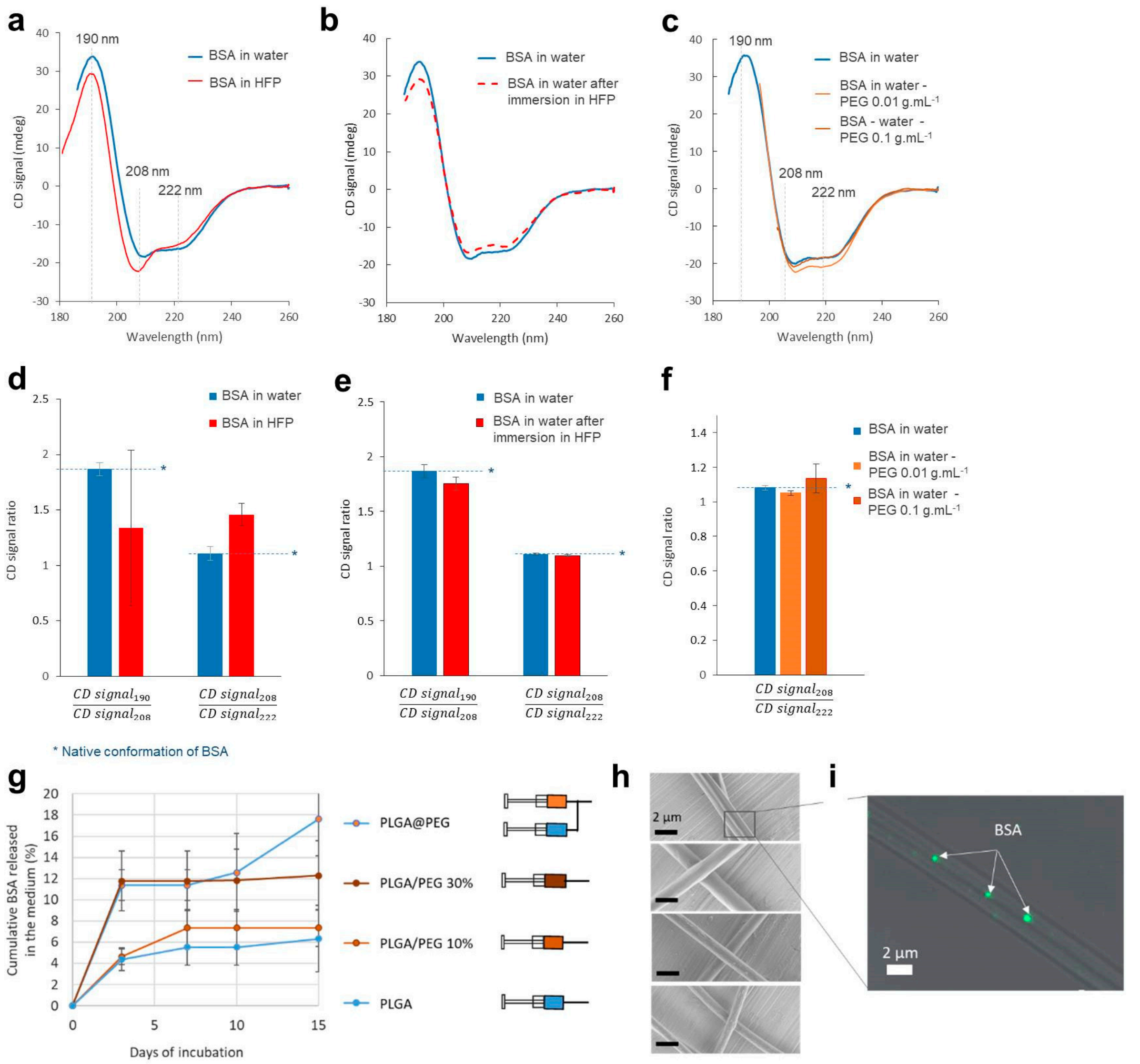

Figure S2 Protein conformation in solution by circular dichroism. a-c) CD spectra of BSA (0.5 mg.mL^{-1}): a) solubilized in water or in HFP, b) in water after immersion of HFP, and c) in water with 0.01 and 0.1 g.mL-1 of PEG; d-f) ratios of CD signal recorded on the corresponding spectra a-c)), respectively, at characteristic wavelengths of 190 and 208 nm, and at 208 and 222; g-i) Kinetics of BSA released from four types of PLGA filaments: g) Cumulative BSA released from filaments made of PLGA, a mixture of PLGA/PEG and by coaxial electrospinning PLGA@PEG. The error bars correspond to the standard deviation obtained from the absorbance measurements. h) SEM images of the four types of filaments loaded with BSA, i) CLSM image of an isolated PLGA@PEG filament loaded with fluorescent BSA.

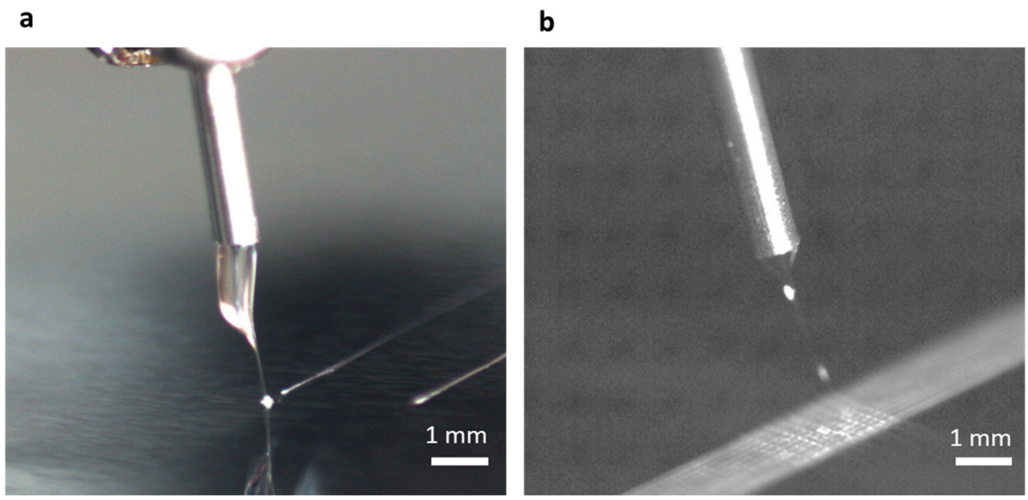

Figure S3. Images of the needle-tip, 5 min after direct-writing a jet of PLGA solution (a) and PCL solution (b)

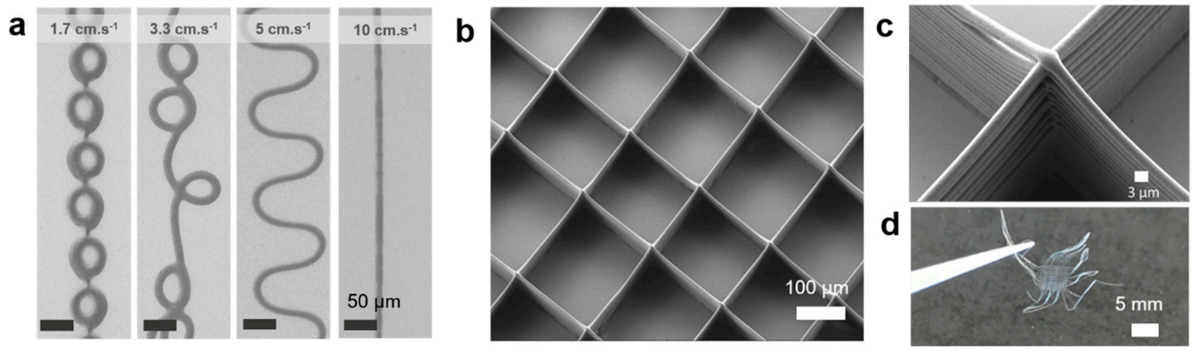

Figure S4. a) Representative SEM images of the pattern formed by the deposited filament depending on the collector speed. b) and c) SEM images of the unfunctionalised PCL scaffold. D) Photo of a PCL scaffold detached from the silicon wafer.

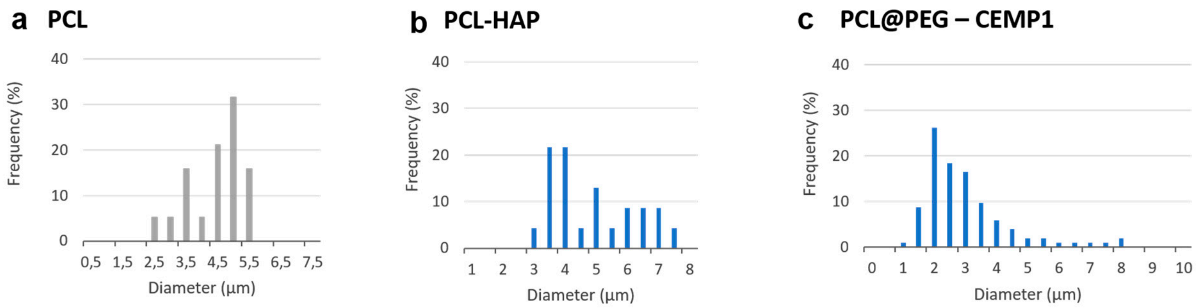

Figure S5. Diameter distribution of filaments composing a) the unfunctionalised PCL scaffold, b) HAP-based scaffold and c) the CEMP1-based scaffold.

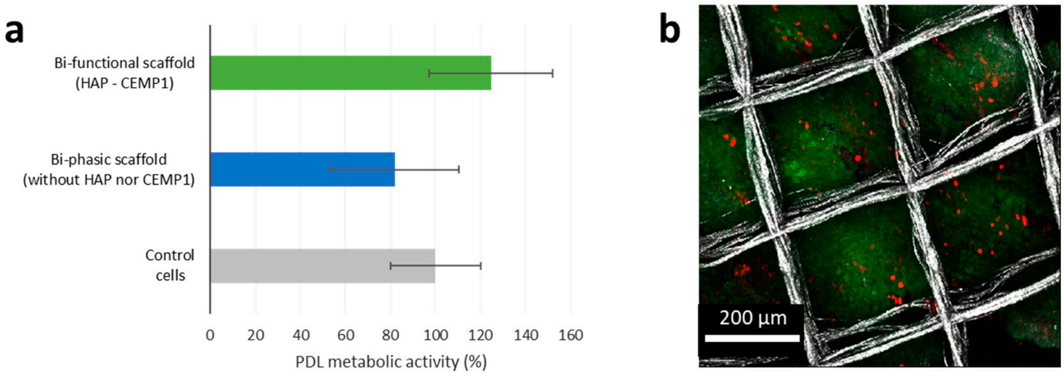

Figure S6. Cytocompatibility assay of the bi-functional scaffold with PDL cells. a) Metabolic activity of cells after 7 days of incubation. b) Fluorescent image of cells with the bi-functional scaffold after staining with a LIVE/DEAD™ assay: living cells stained in green, damaged cells in red, and the scaffold appears in gray.

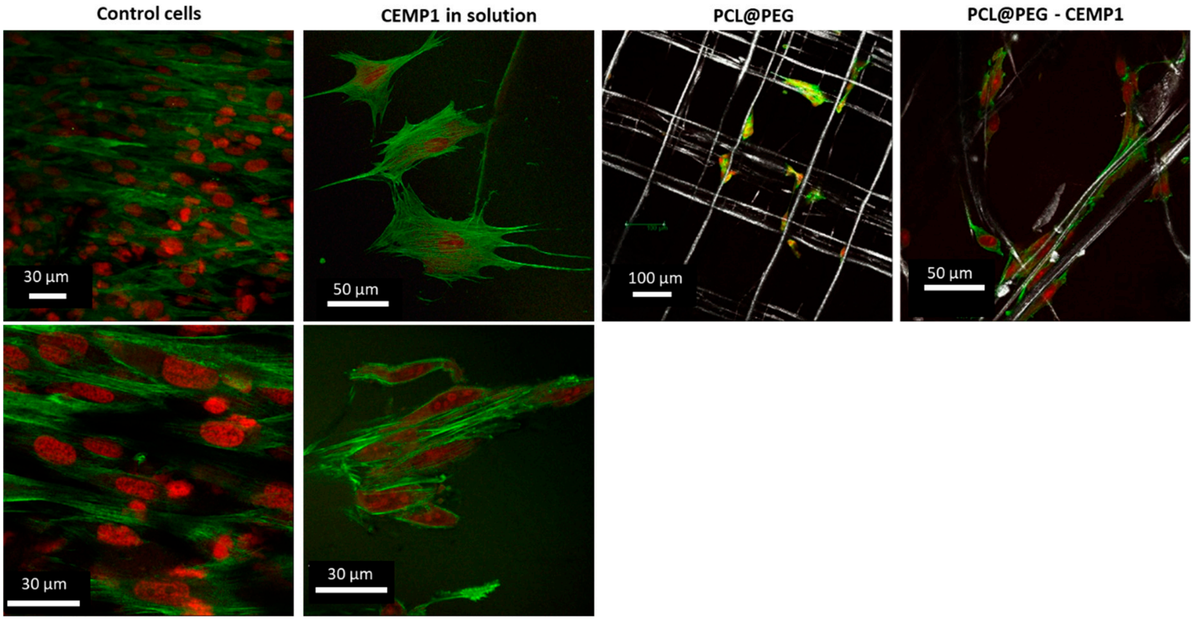

Figure S7. CLSM images of PDL cells after 7 days of incubation in contact with CEMP1-based scaffolds. The cell nuclei were stained with PI (in red) and the actin filaments were stained with phalloidin Alexa 488 (in green).

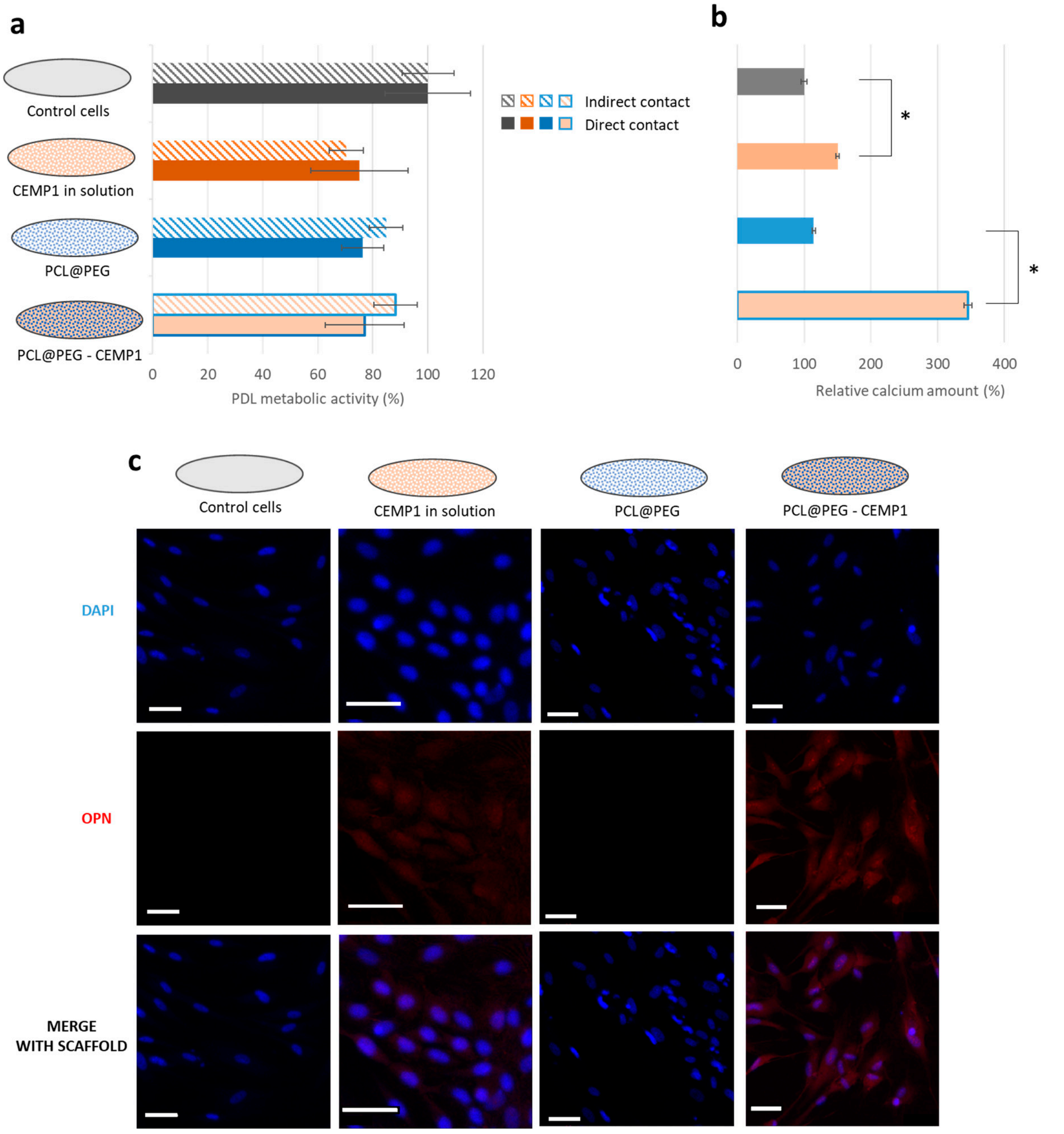

*Figure S8. Assessment of PDL cell behavior in indirect contact with the CEMP1-based scaffold. a) The cellular metabolic activity rate determined after 7 days from direct and indirect contact. b) Relative amount of calcium stained by Alizarin S red. *P < 0.001. c) CLSM images taken after cell nuclei staining with DAPI (in blue) and OPN staining (in red) with an anti-osteopontin conjugated with AF555 (scale bars are 50 μm).*
