# Supplementary material for: Direct-Writing Electrospun Functionalized Scaffolds for Periodontal Regeneration: In Vitro Studies
Source: J Funct Biomater. 2023 May 9;14(5):263. doi: 10.3390/jfb14050263 (PMC10218971; doi:10.3390/jfb14050263)
Supplement: Supplementary file 1 [file jfb-14-00263-s001.zip › Supplementary material S1.pdf]

SUPPLEMENTARY MATERIAL S1: OPTIMIZATION OF SCAFFOLDS CONTAINING PROTEINS

Direct-Writing Electrospun of Functional Scaffolds for Periodontal Regeneration: In Vitro Studies

Laura Bourdon[1], Nina Attik[1,2], Liza Belkessam[1,2], Charlène Chevalier[1,2], Colin Bousige[1], Arnaud Brioude[1] and Vincent Salles[1,3,4*]

[1] Laboratoire des Multimatériaux et Interfaces, UMR 5615, CNRS, Université Claude Bernard Lyon 1, Bâtiment Chevreul, 6 rue Victor Grignard, 69622 Villeurbanne, France

[2] Faculté d'Odontologie, Université Lyon 1, 11 Rue Guillaume Paradin, 69008 Lyon, France

[3] LIMMS, CNRS-IIS UMI 2820, The University of Tokyo, Tokyo 153-8505, Japan

[4] Institute of Industrial Science, The University of Tokyo, Tokyo 153-8505, Japan

C1 - Internal use

CONFORMATION OF BSA WITH HFP AND PEG

We studied the protein conformation in solution by circular dichroism (CD, Chirascan spectrophotometer, Applied Photophysics). As CEMP1 is expensive, this part of the study was conducted with BSA protein. The latter can have a different structural sensitivity compared to CEMP1 but, on the other hand, is largely studied in the literature as a good model protein for electrospinning and for conformation change [1–4]. CD spectra were compared based on the intensities of the characteristic peaks at 190, 208 and 222 nm [5]. CD spectra of BSA (0.05 mg.mL^{-1}) solubilized in water or in HFP showed differences suggesting a modification of the BSA conformation caused by the presence of HFP (Fig. S2a). As the CD signal is dependent on the protein concentration, two ratios of the CD signal intensity at characteristic wavelengths were used for a clear comparison without standardization. First, there is the ratio of the two highest CD peaks at 190 and 208 nm, then the ratio of CD signals at 208 and 222 nm which can be used to characterize the proportion of α-helix [5]. The peak intensities at 222 nm in water and in HFP are similar but the ones at 190 and 208 nm are significantly different by around 30% (Fig. S2d). Those last two peaks being associated with the α-helix conformation, the difference suggests a slight change of α-helix into β-sheet or random conformation. However, by putting the protein immersed in HFP back into the water, the CD signal tends to return to that of the BSA in water (Fig. S2b and e). This phenomenon highlights a certain reversibility of the conformation change. However, in order to see if using a core-shell approach could allow protecting more effectively the conformation of the protein, the CD signal of solutions containing the protein and a water soluble polymer was investigated (Fig. S2c and f). We confirmed that PEG in water had no effect on this conformation, or at least until a concentration of 10wt%.

Encapsulation and release of BSA from PLGA filaments

C1 - Internal use

The BSA was encapsulated in the core of the core-shell PEG-PLGA filament. A solution of 0.1 g.mL^{-1} of PLGA in HFP and a solution of 0.1 g.mL^{-1} of PEG with 20 mg.mL^{-1} of BSA in water were prepared and extruded through coaxial needles of 28G and 21G with a flow rate of 0.12 and 0.5 mL.h^{-1}, respectively. The filaments were spun with a voltage of 5 kV and a 10 cm working distance. To confirm the encapsulation of BSA, the core-shell filaments with fluorescent BSA at 0.5 mg.mL^{-1} (BSA-Alexa Fluor^{TM} 488 conjugate protein, ThermoFisher) were observed by confocal microscopy (Zeiss LSM 800) (Fig. S2i). After drying and sterilization, the filaments were incubated in water at 37°C for 6 days. The amount of BSA in the medium was measured after 3, 7, 10 and 15 days by spectrophotometry (absorbance at 280 nm by NanoDrop^{TM}).

1 References

1. Jiang, H.; Hu, Y.; Li, Y.; Zhao, P.; Zhu, K.; Chen, W. A Facile Technique to Prepare Biodegradable Coaxial Electrospun Nanofibers for Controlled Release of Bioactive Agents. *J. Control. Release* **2005**, *108*, 237–243, doi:10.1016/j.jconrel.2005.08.006.

2. Qi, H.; Hu, P.; Xu, J.; Wang, A. Encapsulation of Drug Reservoirs in Fibers by Emulsion Electrospinning: Morphology Characterization and Preliminary Release Assessment. *Biomacromolecules* **2006**, *7*, 2327–2330, doi:10.1021/bm060264z.

3. Moriyama, Y.; I, K.A.; Takeda, K.; Wada, A.; Yamamoto, K. Conformational Change of Bovine Serum Albumin by Heat Treatment. *J. Protein Chem.* **1989**, *8*, 653–659.

4. He, S.; Huang, M.; Ye, W.; Chen, D.; He, S.; Ding, L.; Yao, Y.; Wan, L.; Xu, J.; Miao, S. Conformational Change of Bovine Serum Albumin Molecules at Neutral PH in Ultra-Diluted Aqueous Solutions. *J. Phys. Chem. B* **2014**, *118*, 12207–12214, doi:10.1021/jp5081115.

5. Kelly, S.M.; Jess, T.J.; Price, N.C. How to Study Proteins by Circular Dichroism. *Biochim. Biophys. Acta* **2005**, *1751*, 119–139, doi:10.1016/j.bbapap.2005.06.005.

C1 - Internal use
